# Finding a needle in a haystack: Evaluation of Cartridge-Based Nucleic Acid Amplification Test in comparison with microscopy and culture for diagnosis of Extrapulmonary Tuberculosis – A study from a tertiary care centre, Kerala

**DOI:** 10.1099/acmi.0.001249.v3

**Published:** 2026-10-20

**Authors:** Benjamin David George, Sreeja Nair

**Affiliations:** 1 Department of Microbiology, Pushpagiri Institute of Medical Sciences and Research Centre, Thiruvalla, KL-689101, India; 2 Associate Professor, Department of Microbiology, Sreemookambika Institute of Medical Sciences, Kulasekharam, TN-629161, India

**Keywords:** CBNAAT, diagnosis, extra-pulmonary tuberculosis, liquid culture, paucibacillary specimen, tuberculosis

## Abstract

**Introduction.** *Mycobacterium tuberculosis* has held an exceptional presence in medical research, especially in recent years (1). While pulmonary infections largely remain the focus of literature and public health initiatives (2), extrapulmonary tuberculosis (EPTB) infections have been gaining prominence due to their increasing prevalence, particularly in populations characterized by a low incidence of tuberculosis (2–4). Although EPTB infections, such as tuberculous meningitis, carry a high mortality risk (5), delays in diagnosis and treatment persist. These delays are driven by their diverse, non-specific clinical presentations, coupled with the variable performance of diagnostic modalities like the Cartridge-Based Nucleic Acid Amplification Test (CBNAAT) (6–10). While general literature regarding the use and efficacy of CBNAAT in diagnosing EPTB exists (2,11–15), studies focusing on its sensitivity and specificity in paucibacillary specimens are an evolving niche (16). Since there is a greater prevalence of paucibacillary EPTB specimens in our setting, where such an analysis has not been conducted, we performed a retrospective study to understand the overarching diagnostic performance of our existing modalities in EPTB specimens.

**Methods.** An observational retrospective study was conducted with the data collected between 2019 and 2022 at a tertiary care centre in central Kerala, India. A total of 105 specimens – categorized as pleural, lymph node, pus, tissue, cerebrospinal fluid (CSF), peritoneal and others – from presumptive EPTB cases were included in the study. The samples underwent acid-fast bacilli staining microscopy, liquid culture and CBNAAT utilizing the GeneXpert platform. The results were recorded, and the data were analysed with *P<*0.05 considered statistically significant.

**Observations and Results.** In the 105 samples collected for the study, 6 (5.7%) were positive on Ziehl–Nielsen staining, 26 (24.8%) samples grew *M. tuberculosis* complex on liquid culture and CBNAAT detected *M. tuberculosis* in 16 (15.2%) specimens. The sensitivity and specificity of CBNAAT, with culture as the gold standard, were found to be 50.0 and 96.2%, respectively. The sensitivity and specificity of CBNAAT for pus, peritoneal (including tissue and fluid aspirate), pleural fluids, CSF, lymph nodes and other miscellaneous sample types ranged from 33.3 to 100% and 85.7 to 100%, respectively. Among smear-negative specimens, the sensitivity and specificity of CBNAAT were 42.9 and 96.2%, respectively, whereas in smear-positive specimens, the sensitivity increased to 80.0%.

**Conclusion.** CBNAAT is a viable tool for the detection of *M. tuberculosis*; however, in paucibacillary specimens, its use along with other methods like smear and culture could lead to better identification of the mycobacteria.

## Data Summary

The authors confirm all supporting data, code and protocols have been provided within the article or in Supplementary Table 1 (avaliable with the online version of this article). The jamovi project (2024). jamovi. (version 2.6) (Computer Software) was used to conduct the statistical analysis of the data obtained from the institutional records.

## Introduction

Ever since its discovery in 1882 by Robert Koch, *Mycobacterium tuberculosis* has held a unique presence in the overarching pursuit of health. The pathogen and the disease it causes have been the subject of study for several researchers over the centuries [1]. Despite advances in the timely identification and treatment of the disease that have led to a substantial decrease in case-based mortality and morbidity, we still grapple with the complete eradication or effective containment of the infection on a global scale [1, 2]. Moreover, the decrease in disease burden has not been equally distributed across all the various infections caused by the organism [2]. While the adverse health outcomes of pulmonary tuberculosis (PTB) infections have been limited due to early detection and initiation of adequate treatment, extrapulmonary tuberculosis (EPTB) infections, such as meningitis, which is characterized by high mortality even today, are a cause of significant concern to clinicians all over the world [3][4].

The challenges in diagnosing the disease begin with the presenting complaints of the patients. Patients suffering from PTB present with distinct clinical features such as fever, persistent nocturnal cough and expectoration lasting more than a week. EPTB, on the other hand, presents primarily with non-specific symptoms and signs associated with the tissue that it affects. For instance, in tuberculous lymphadenitis, the patient presents with complaints of swelling in the region of the infected lymph nodes, along with fever lasting from months to years [5, 6]. This dichotomy consumes valuable clinical hours to rule out other pathogens or illnesses while the disease progresses.

Similarly, the diagnostic accuracy of standard microbiological modalities used to confirm EPTB differs from that of PTB . Although methods such as light microscopy using acid-fast staining, liquid culture and Cartridge-Based Nucleic Acid Amplification Test (CBNAAT) using GeneXpert system (manufactured by Cepheid, Sunnyvale, CA, USA) prove effective in identifying the micro-organism, their performance with extrapulmonary specimens is not as well defined [7, 8]. In studies with a lower proportion of paucibacillary specimens, such as pleural fluids or cerebrospinal fluids (CSFs), these investigative tools performed well; however, when these specimen types were studied exclusively, their utility was limited [7, 9–12]. This variation compounds the delay in resolving clinical suspicion, resulting in delayed initiation of much-needed treatment for the patients.[13]

All these factors, along with microbial resistance to treatment and non-compliance with an arduous regimen, make EPTB a disease of great concern to healthcare systems [14]. Therefore, addressing the diagnostic efficacy of existing microbiological tools could bridge one of the pivotal gaps in achieving prompt management of the disease. There exists literature regarding the efficacy of CBNAAT, using the GeneXpert platform, with respect to various EPTB specimens, such as lymph nodes, pleural fluids, CSF, peritoneal and other specimens, either combined or exclusively [15–20]. In most studies, the predominant specimen type among those included was lymph node [17, 19, 21–25], reflecting the prevalence of EPTB in the region where these studies were conducted. The results of these studies demonstrate a consensus that CBNAAT offers comparable or superior sensitivity and specificity for lymph node samples in identifying the pathogen compared to liquid culture. However, studies with predominant paucibacillary specimens have shown lower sensitivity and specificity of CBNAAT for these samples [25]. Even studies that assessed the performance of CBNAAT in various paucibacillary samples concur with these findings [17, 24]. Such studies have not been conducted in our setting, where there is a greater prevalence of paucibacillary specimens. Considering the rapid development in diagnostic devices and methods, it is essential to understand the capabilities and limitations of existing modalities within our setting to help us advance local tuberculosis mitigation strategies [12, 26].

## Methods

An observational retrospective study was conducted in the laboratory of the Department of Microbiology, Pushpagiri Institute of Medical Sciences and Research Centre, Thiruvalla, Kerala, India, following approval from the Institutional Ethics Committee (IEC No. PIMSRC/E1/388A/14/2022) and general informed consent from the participants. CBNAAT was performed at the District Tuberculosis Centre (DTC), Pathanamthitta, Kerala, India, and the reports were delivered to the laboratory in the department.

A total of 105 samples from the historical data collected between 2019 and 2022 were included in the study, which was conducted over a 2-month period (August 2022 to September 2022). All presumptive EPTB cases irrespective of age, previous history of anti-tubercular treatment (ATT) – provided they were not on ATT at the time of sampling – were included in the study. The diagnosis of EPTB was made if acid-fast bacilli (AFB) were detected in the smears stained by the Ziehl–Neelsen (ZN) method, *M. tuberculosis* growth was observed on liquid culture in automated Baltimore Biological Laboratory (BBL) Mycobacteria Growth Indicator Tube (MGIT; BD BACTEC, BD Biosciences, Sparks, MD, USA) or *M. tuberculosis* nucleic acid was identified by CBNAAT using the GeneXpert platform in any clinical specimen.

Specimens were collected in sterile containers with tight-fitting lids and transported to the laboratory with minimal delay. They were then processed using the *N*-acetyl-l-cysteine and sodium hydroxide (NALC–NaOH) technique for digestion and decontamination [27]. This process was omitted only for sterile body fluids such as CSF.

Once the smears were prepared and stained following the ZN staining method, AFB were identified by their typical slender, rod-shaped, distinct morphology against the methylene blue background. The smears were reported positive in accordance with the National Tuberculosis Elimination Programme guidelines [28].

The samples for culture in the automated BBL MGIT system were processed according to the manufacturer’s guidelines and inoculated [27]. The tubes were incubated and inspected manually every day, starting 10 days post-inoculation, for the presence of granular appearance and fluorescence indicative of growth. *M. tuberculosis* complex (MTBc) isolates were confirmed using the BD MGIT MTBc identification test (TBc ID) according to the manufacturer’s instructions [27].

Samples for CBNAAT were processed per the instructions of the manufacturer and sent to the DTC, Pathanamthitta, Kerala, India. The samples were analysed on the GeneXpert platform and the reports updated to the hospital database.

The results along with patient details such as age, sex and associated comorbidities were recorded, tabulated and then analysed using standard statistical methods (jamovi, version 2.6; The jamovi project, 2024). Patient parameters such as comorbidities were computed, and their proportions in the sample were assessed using Bayesian binomial test, with *P<*0.05 considered statistically significant. The sensitivity, specificity, positive predictive value (PPV), negative predictive value (NPV) and diagnostic accuracy of CBNAAT, with liquid culture as the reference gold standard, were calculated for all the specimens, and the results were compared.

## Observations and results

An observational retrospective study was conducted in a tertiary care centre in central Kerala, India, after obtaining clearance from the IEC and consent from patients. The study was over a period of 2 months (August 2022 to September 2022) and included the data of 4 years (2019–2022). For the study, a total of 105 samples were collected. The mean age of the study population was 48.8 (±19.43) years with the mean age of the male sex (*n*=60) being 53.3 (±19.2) years and that of the female sex (*n*=45) being 42.8 (±18.3) years (Fig. 1). The mean age difference was found to be statistically significant (*t*(103)=2.83, *P=*0.006), with moderate Bayesian evidence (BF_10_=6.80) supporting this finding (Table 1). The most common comorbidity was hypertension in 40.6% of the individuals followed by diabetes mellitus in 39.6% of the study population. The proportion of males who were positive (29.6%) was significantly higher than that of females (21.42%) (*χ*^2^=40.6, *P*<0.001).

**Fig. 1.**
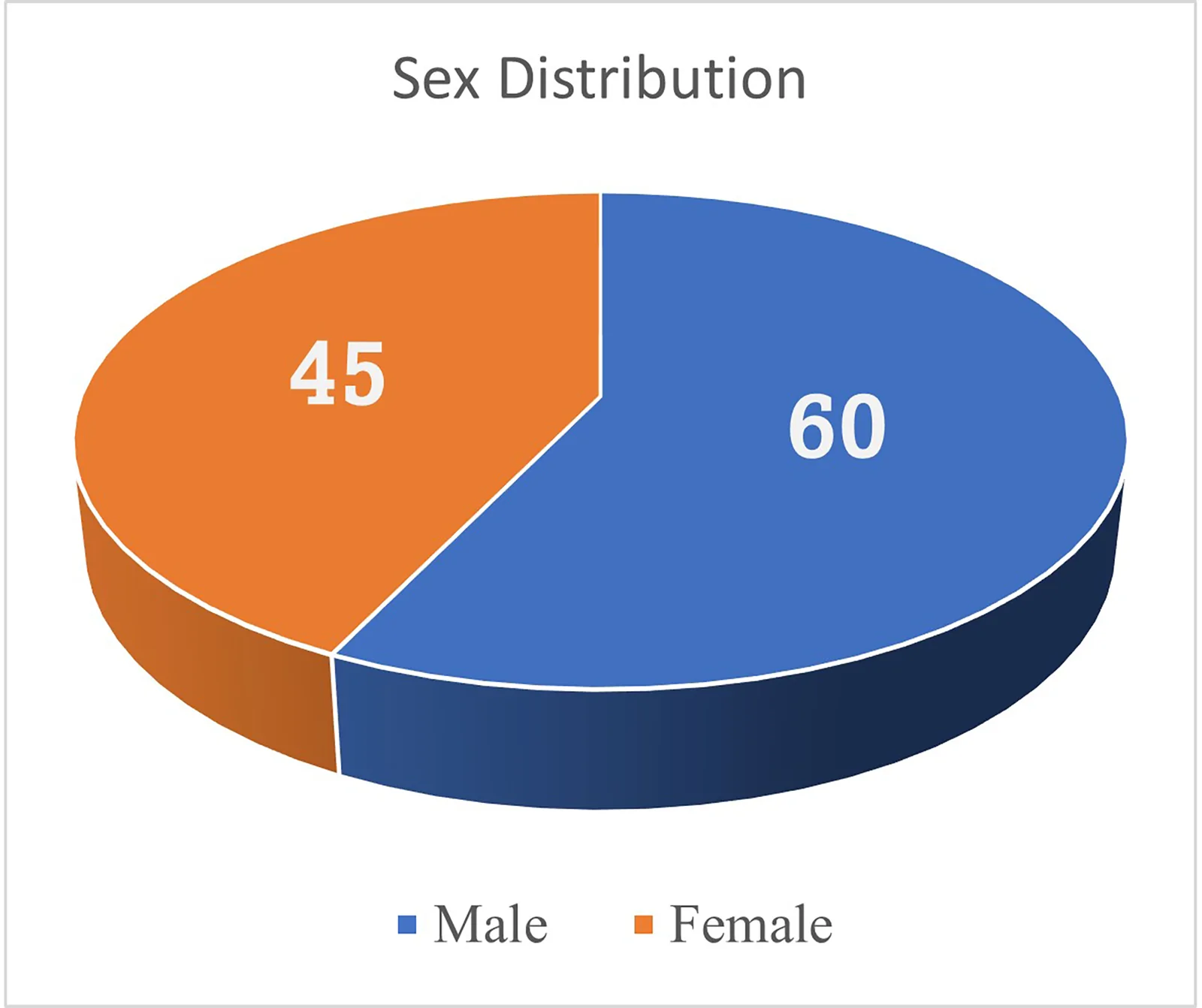
Sex distribution of patients.

**Table 1.** Distribution of study sample based on gender

	Sex	*N*	Proportion (%)	Mean	95% confidence interval		
					Lower	Upper	sd
**Age**	Male	60	57.1	53.3	48.3	58.2	19.2
	Female	45	42.9	42.8	37.3	48.2	18.3

* The confidence interval of the mean assumes that sample means follow a *t*-distribution with *N−*1 degrees of freedom.

Of the 105 specimens, 6 (5.7%, *P*<0.001) were positive for AFB on ZN staining, 26 [24.8%, 95% confidence interval (CI): 16.9, 34.1, *P*<0.001] grew MTBc on liquid culture, and 16 (15.2%, *P*<0.001) had *M. tuberculosis* nucleic acid detected by CBNAAT. Of all the samples analysed by CBNAAT, only *n*=1 (1%) each were categorized as ‘high’ and ‘medium’ bacterial loads, while the remainder of positive samples were categorized as ‘low’ (*n*=14, 13.3%, *P<*0.001).

The sensitivity of microscopy (AFB smear), keeping culture as the gold standard, was 19.2% (95% CI: 6.6, 39.4), and the specificity 98.7% (95% CI: 93.1, 100) (Table 2).

**Table 2.** Diagnostic characteristics of smear examination in EPTB

Smear	Culture		Total
	Growth present	No. of growth	
AFB seen	5	1	6
AFB not seen	21	78	99
Total	26	79	105

Diagnostic accuracy = (TP+TN)/(TP+TN+FP+FN) = 79.0% (95%CI: 70.0, 86.4).

Positive predictive value = TP/(TP+FP) = 83.3% (95%CI: 35.9,99.6).

Negative predictive value = TN/(TN+FN) =78.8% (95%CI: 69.4, 86.4).

Sensitivity = TP/(TP+FN) = 19.2% (95%CI:6.6,39.4).

Specificity = TN/(TN+FP) = 98.7% (95%CI: 93.1,100.0).

The most common type of specimen was pleural (*n*=34, 32.7%) with *M. tuberculosis* growth in three (2.9%), followed by lymph nodes (*n*=20, 19.2%) with growth in seven (6.7%) of the samples. Various tissues (*n*=12, 11.4%) had growth in 2 (1.9%) of the samples, while 5 (4.8%) of the 12 pus samples (11.4%) had *M. tuberculosis* growth. Of the ten peritoneal samples (9.5%; including tissue biopsy and ascitic fluid), five (4.8%) were positive for growth of *M. tuberculosis*. Three (2.9%) of the eight (7.6%) CSF specimens showed growth, and one (1.0%) of the nine (8.6%) other fluid samples (which includes synovial fluid, aspirate fluid, aspirate abscess and urine) showed growth on culture (Fig. 2, Table 3).

**Fig. 2.**
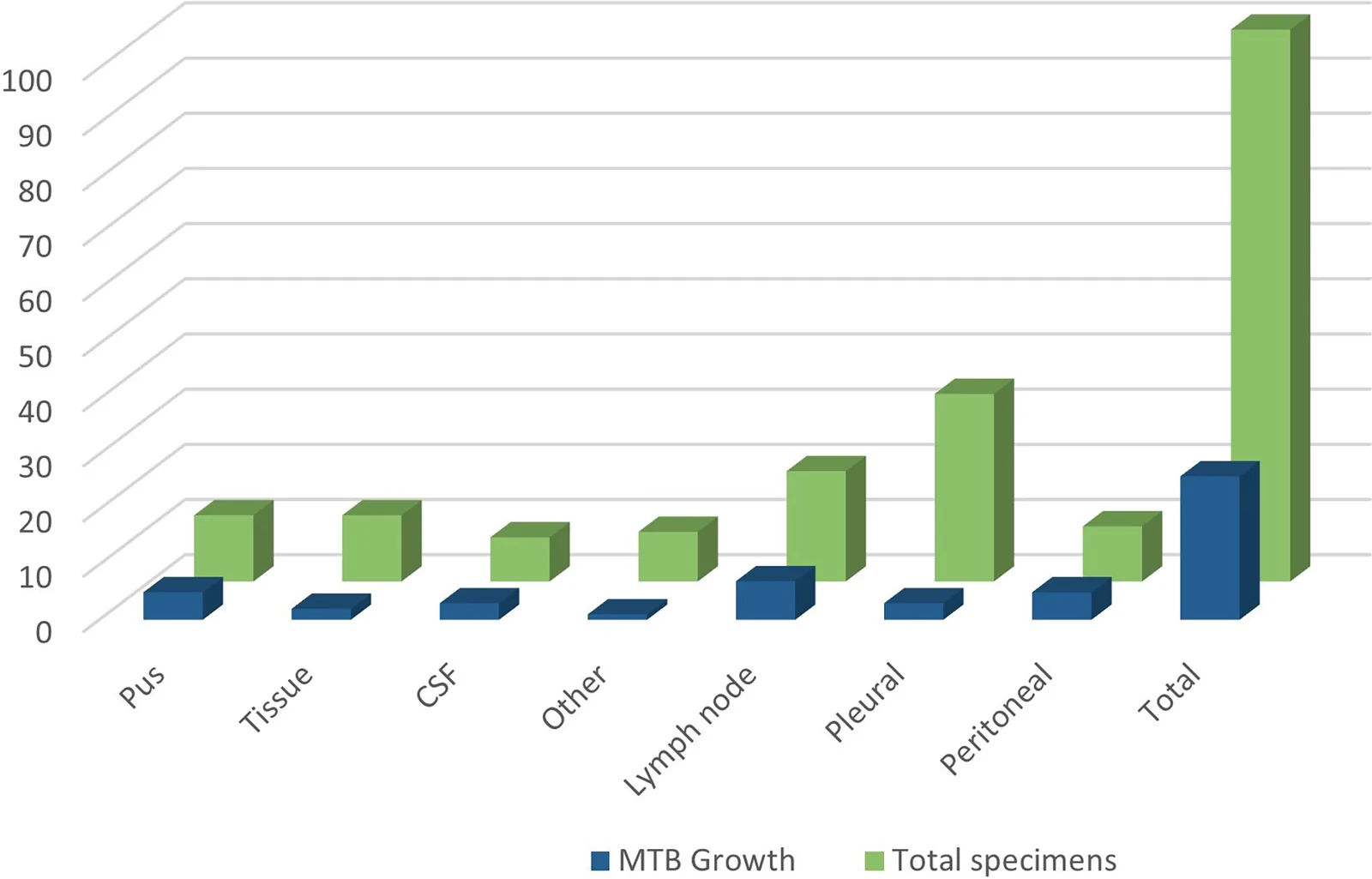
Distribution of *M. tuberculosis* growth among specimens.

**Table 3.** Distribution of study specimen

Liquid culture		Pus	Tissue	CSF	Other	Lymph node	Pleural	Peritoneal	Total
Negative	Observed	7	10	5	8	13	31	5	79
	% of total	6.7	9.5	4.8	7.6	12.4	29.5	4.8	75.2
Positive	Observed	5	2	3	1	7	3	5	26
	% of total	4.8	1.9	2.9	1.0	6.7	2.9	4.8	24.8
Total	Observed	12	12	8	9	20	34	10	105
	% of total	11.4	11.4	7.6	8.6	19.0	32.4	9.5	100.0

Fisher’s exact test; *P=*0.026.

Fisher’s exact test was performed to assess the relationship between specimen type and liquid culture, as the sample count across individual types was expected to be low. It found that the outcomes varied across types of specimens significantly (Fisher’s exact test, *P*=0.026, Cramer’s *V*=0.352). The sample size (*N*=105) was considered moderate to large, and the post-hoc analysis of standardized residuals showed that pleural fluid specimens had no growth on culture more often than expected (*z*=2.62), while growth was observed in peritoneal samples more often than expected (*z*=1.94), approaching the statistical significance threshold of 1.96 (Table 3).

The pooled sensitivity of CBNAAT with culture as the gold standard was found to be 50.0% (95% CI: 29.9, 70.1) and the specificity 96.2% (95% CI: 89.3, 99.2). The PPV was 81.3% (95% CI: 54.4, 96.0) and the NPV 85.4% (95% CI: 76.3, 92.0). According to our study, the diagnostic accuracy for CBNAAT was 84.8% (95% CI: 76.4, 91.0). (Table 4).

**Table 4.** Diagnostic characteristics of CBNAAT

CBNAAT	Culture		Total
	Growth present	No. of growth	
Detected	13	3	16
Not detected	13	76	89
Total	26	79	105

Sensitivity = TP/(TP+FN) = 50.0% (95%CI:29.9,70.1).

Specificity = TN/(TN+FP) = 96.2% (95%CI:89.3,99.2).

Positive predictive value = TP/(TP+FP) =81.3% (95%CI:54.4,96.0).

Negative predictive value = TN/(TN+FN) = 85.4% (95%CI:76.3,92.0).

Diagnostic accuracy = (TP+TN)/(TP+TN+FP+FN) = 84.8% (95%CI:76.4, 91.0).

Stratification of the sensitivity and specificity of CBNAAT for each specimen category revealed widely dispersed values. For instance, the sensitivity and specificity for pus samples were 80% (95% CI: 28.4, 99.5) and 85.7% (95% CI: 42.1, 99.6), respectively. However, for peritoneal samples, it was 20% (95% CI: 0.5, 71.6) and 80% (95% CI: 28.4, 99.5), respectively. Interestingly, pleural fluids and CSF had the same sensitivity (33.3%; 95% CI: 0.8, 90.6), but the specificity for the former was 100% (95% CI: 88.8, 100) and the latter was 100% (95% CI: 47.8, 100). The sensitivity for lymph nodes was 71.4% (95% CI: 29, 96.3) and specificity 92.3% (95% CI: 64, 99.8), while in the case of other fluids, it was 100% (95% CI: 2.5, 100) sensitive and 100% (95% CI: 63.1, 100) specific (Table 5). The tissue samples (*n*=12) could not be evaluated, as both the samples that grew *M. tuberculosis* on liquid culture were reported as negative for *M. tuberculosis* nucleic acid on CBNAAT. The diagnostic accuracy for the various specimen types ranged from 50% (95% CI: 18.7, 81.3) to 100% (95% CI: 66.4, 100), giving a total accuracy of 84.8% (95% CI: 76.4, 91).

**Table 5.** Comparison of diagnostic characteristics of CBNAAT in various categories of specimen

Diagnostic characteristics	Pus (%)	Pleural fluid (%)	CSF (%)	Lymph node (%)	Peritoneal (%)	Other fluids^*^ (%)	Overall (%)
Sensitivity (95% CI)	80 (28.4, 99.5)	33.3 (0.8, 90.6)	33.3 (0.8, 90.6)	71.4 (29, 96.3)	20 (0.5, 71.6)	100 (2.5, 100)	50 (29.9, 70.1)
Specificity (95% CI)	85.7 (42.1, 99.6)	100 (88.8, 100)	100 (47.8, 100)	92.3 (64, 99.8)	80 (28.4, 99.5)	100 (63.1, 100)	96.2 (89.3, 99.2)
PPV (95% CI)	85.7 (42.1, 99.6)	100 (2.5, 100)	100 (2.5, 100)	83.3 (35.9, 99.6)	50 (1.3, 98.7)	100 (2.5, 100)	81.3 (54.4, 96)
NPV (95% CI)	80 (28.4, 99.5)	93.9 (79.8, 99.3)	71.4 (29, 96.3)	85.7 (57.2, 98.2)	50 (15.7, 84.3)	100 (63.1, 100)	0.854 (76.3, 92)
Diagnostic accuracy (95% CI)	83.3 (51.6, 97.9)	94.1 (80.3, 99.3)	75 (34.9, 96.8)	85 (62.1, 96.8)	50 (18.7, 81.3)	100 (66.4, 100)	84.8 (76.4, 91)

*Other fluids include synovial fluid, aspirate fluid, aspirate abscess and urine.

Among smear-negative specimens (*n*=99), the sensitivity, specificity, PPV and NPV of CBNAAT were 42.9% (95% CI: 21.8, 66.0), 96.2% (95% CI: 89.2, 99.2), 75.0% (95% CI: 42.8, 94.5), 86.2% (95% CI: 77.1, 92.7) and 84.8% (95% CI: 76.2, 91.3), respectively (Table 6). In smear-positive specimens, the sensitivity increased to 80.0% (95% CI: 28.4, 99.5) (Table 7).

**Table 6.** Diagnostic criteria of CBNAAT in the smear-negative specimen

Smear-negative specimen	Culture		Total
CBNAAT	Growth	No. of growth	
Detected	9	3	12
Not Detected	12	75	87
Total	21	78	99

Sensitivity=TP/(TP+FN)=42.9% (95% CI: 21.8, 66.0).

Specificity=TN/(TN+FP)=96.2% (95% CI: 89.2, 99.2).

Diagnostic accuracy=(TP+TN)/(TP+TN+FP+FN)=84.8% (95% CI: 76.2, 91.3).

PPV=TP/(TP+FP)=75.0% (95% CI: 42.8, 94.5).

NPV=TN/(TN+FN)=86.2% (95% CI: 77.1, 92.7).

**Table 7.** Diagnostic characteristics of CBNAAT in smear-positive specimens

Smear-positive specimen	Culture		Total
CBNAAT	Growth	No. of growth	
Detected	4	0	4
Not Detected	1	1	2
Total	5	1	6

Sensitivity=TP/(TP+FN)=80.0% (95% CI: 28.4, 99.5).

Specificity=TN/(TN+FP)=100.0% (95% CI: 2.5, 100.0).

## Discussion

Given that tuberculosis has a plethora of clinical features [17, 22, 29] when infecting extrapulmonary tissues, it is imperative that a method of microbiological diagnosis be developed to detect the organism. To this end, organizations like the World Health Organization have issued guidelines [30, 31] on how to diagnose the condition in various settings.

For the present study, a pool of 105 samples was collected, and it was found that the proportions of male and female sexes within it were insignificantly different. However, when factoring in the age distribution of the sexes, it could be inferred that a younger female population demonstrated health-seeking behaviour for their symptoms, while a significant proportion of men were diagnosed with the disease, consistent with the findings of other studies conducted in Kerala [32, 33] and internationally [34]. This also corresponds to the data provided by the studies on sex and EPTB [35]. This could be explained by the higher literacy rate of the general population in the region.

While smear preparation and staining are among the fastest diagnostic tools, their performance when compared to culture showed a sensitivity of 19.2% (95% CI: 6.6, 39.4) and a specificity of 98.7% (95% CI: 93.1, 100), which is congruent with other similar studies and suggests the combined use of smear with other diagnostic tools such as CBNAAT or culture.

The assessment of the performance of CBNAAT, with liquid culture as the gold standard, found the sensitivity to be 50.0% (95% CI: 29.9, 70.1) and the specificity 96.2% (95% CI: 89.3, 99.2). This is similar to other studies [36, 37], where CBNAAT demonstrated lower performance with extrapulmonary specimens than pulmonary samples within the same study. It could be observed that the composition of the sample and the presence of paucibacillary specimens, such as pleural or CSF specimens [10], could have an impact on the overall diagnostic accuracy of the test or that the decontamination of pleural specimens with NALC–NaOH could have been too harsh, resulting in no growth [17]. However, it must be highlighted that, in the case of lymph node specimens, the performance of CBNAAT remained comparable to existing literature studies [7, 26, 38], confirming the established utility of the system in reaching a diagnosis.

The impact of this composition on the overall diagnostic accuracy of CBNAAT was further examined, considering that pleural samples contributed the most (*n*=34, 32.4%) to the total sample pool and that CBNAAT was 100% specific but only 33.3% sensitive against culture. Utilizing the post-hoc analysis of standardized residuals, it was confirmed that pleural samples yielded negative cultures significantly more often than expected (*z*=2.62, *P*=0.026). This leads to the inference that CBNAAT is best used with other diagnostic modalities, such as culture, to ensure effective patient care. This also emphasizes the need for studies on the diagnostic accuracy of various methods like CBNAAT in paucibacillary specimens, since there remain challenges in diagnosing EPTB clinically and it carries high mortality and morbidity rates [39].

Another significant element in the measures of diagnostic accuracy of CBNAAT in our study is the false-positive results. While the GeneXpert platform (CBNAAT) is capable of detecting DNA that is in minimal quantities (100–130 bacilli ml^–1^) within specimens [26], the viability of this nucleic acid is a critical clinical determinant in the management of the patient. If it is dormant or non-viable DNA, the challenge to initiating ATT for the patient rests on the risk of adverse effects like hepatotoxicity and its sequelae [40].

When considering the performance of CBNAAT in smear-negative and smear-positive specimens, it could be stated that CBNAAT performs better in smear-positive specimens [8, 41]. However, the small sample size and wide confidence intervals for the specificity in smear-positive specimens (*n=*6) make it challenging to draw a statistically significant conclusion.

Therefore, further research with larger sample sizes and proportion of smear-positive paucibacillary specimens needs to be done in the future.

## Conclusion

This study assessed the performance of CBNAAT against that of liquid culture and has reached the following conclusions:

- CBNAAT is a viable tool for the detection of *M. tuberculosis,* with the pooled sensitivity of 50.0% (95% CI: 29.9, 70.1) and specificity of 96.2% (95% CI: 89.3, 99.2).
- The performance of CBNAAT in paucibacillary specimens, such as pleural fluid, CSF and peritoneal specimens, was inferior to that in multibacillary specimens, like lymph nodes, validating its use along with other methods, such as smear and culture, to yield better results in the identification of mycobacteria.
- Moreover, CBNAAT demonstrated a superior sensitivity of 80.0% (95% CI: 28.4, 99.5) in identifying the disease in smear-positive specimens compared to the smear-negative cohort.

This study highlights the need to look at EPTB, not as a homogeneous entity, but with an approach that factors in the type of specimens involved, utilizing adequate diagnostic modalities to confirm the microbiological diagnosis. In our setting, CBNAAT is useful in diagnosing patients. It would be enhanced if supplemented by other diagnostic tools, such as liquid culture and smear staining, especially for paucibacillary specimens. However, the study is limited by its small sample size, prompting the need for future studies involving larger sample sizes and more diverse groups of specimens to expand on the conclusions, limitations and trends observed in this study.

## Supplementary material

10.1099/acmi.0.001249.v3 Table S1.

## Abbreviations

AFB: acid-fast bacilli
ATT: anti-tubercular treatment
BBL: Baltimore Biological Laboratory
CBNAAT: Cartridge-Based Nucleic Acid Amplification Test
CI: confidence interval
CSF: cerebrospinal fluid
DTC: District Tuberculosis Centre
EPTB: extrapulmonary tuberculosis
FN: false negative
FP: false positive
ICMR: Indian Council of Medical Research
IEC: Institutional Ethics Committee
MGIT: Mycobacteria Growth Indicator Tube
MTBc: *M. tuberculosis* complex
NALC–NaOH: *N*-acetyl-l-cysteine and sodium hydroxide
NPV: negative predictive value
PPV: positive predictive value
PTB: pulmonary tuberculosis
STS: Short-Term Studentship
TN: true negative
TP: true positive
ZN: Ziehl–Neelsen
